# Exploring Angiotensin II Receptor Blockers as a potential protective therapeutic for the Preclinical Stages of Alzheimer's Disease

**DOI:** 10.1002/alz70858_102226

**Published:** 2025-12-25

**Authors:** Julia L Waszak, Gabriel Gach, Carson Hovey, Nicholas Martin, Michael Zawada

**Affiliations:** ^1^ Montana College of Osteopathic Medicine, Billings, MT, USA

## Abstract

**Background:**

There has been a growing interest in providing pharmacological therapy for the stage in Alzheimer's Disease (AD) where there are no noticeable cognitive symptoms, but pathological changes begin. This stage is defined as Preclinical AD. Monoclonal antibody drugs are being developed to provide disease‐modifying effects in Preclinical AD. There is a need for an option that is easily administered, cost‐effective, and widely available that can provide potential neuroprotective benefits. Angiotensin II Receptor Blockers (ARBs) are a potential protective therapeutic for Preclinical AD through anti‐inflammatory and vasodilatory pathways which can clear Ab in the brain, reducing plaque formation. This review explores what is known about ARBs in AD through various well‐known clinical trials while commenting on their potential use as a therapeutic in Preclinical AD.

**Method:**

Performed literature searches in databases such as PubMed, NIH Clinical Trials, NEJM, and JAMA using keywords such as “Alzheimer's Disease”, “Preclinical”, and “ARBs”.

**Result:**

A select few studies are well‐established and published in notable journals that describe the use of ARBs in cognitively normal (CN) older adults and symptomatic AD. The CN studies, Study on Cognition and Prognosis in the Elderly (SCOPE), and ONTARGET/TRANSCEND were among the first studies to investigate the neuroprotective effects of ARBs as secondary endpoints. The symptomatic AD studies “Reducing the pathology of AD through Angiotensin Targeting” (RADAR) and “Candesartan's effects on AD and related biomarkers” (CEDAR) use current methods in exploring the potential efficacy of ARBs in symptomatic AD. CEDAR showed reduced Ab accumulation in groups treated with candesartan.

**Conclusion:**

Disease‐modifying effects have been observed from monoclonal antibodies in symptomatic AD clinical trials. These are now being tested in Preclinical AD. ARBs may provide additional neuroprotective effects. It is also an appropriate option for those who don’t have the economic or geographic means to receive monoclonal antibody infusions. With novel blood biomarkers for tau and Ab, Preclinical AD can be more easily diagnosed in the future. If this is the case, then perhaps prescribing an ARB preliminarily could slow any early pathological process occurring. In addition to a monoclonal antibody, the effects could be even more pronounced.

